# Oral *Macrocystis pyrifera* Fucoidan Administration Exhibits Anti-Inflammatory and Antioxidant Properties and Improves DSS-Induced Colitis in C57BL/6J Mice

**DOI:** 10.3390/pharmaceutics14112383

**Published:** 2022-11-04

**Authors:** Tauseef Ahmad, Muhammad Ishaq, Samuel Karpiniec, Ahyoung Park, Damien Stringer, Neeraj Singh, Vishal Ratanpaul, Karen Wolfswinkel, Helen Fitton, Vanni Caruso, Rajaraman Eri

**Affiliations:** 1 College of Health and Medicine, University of Tasmania, Newnham, TAS 7248, Australia; 2 School of Pharmacy and Pharmacology, University of Tasmania, Hobart, TAS 7001, Australia; 3 Marinova Pty Ltd., Cambridge, TAS 7170, Australia; 4 School of Science, RMIT University, Bundoora West Campus, Plenty Road, Melbourne, VIC 3083, Australia; 5 Department of Pathology, Launceston General Hospital (LGH), Launceston, TAS 7250, Australia; 6 RDadvisor, Hobart, TAS 7006, Australia; 7 Istituto di Formazione e Ricerca in Scienze Algologiche (ISAL), Torre Pedrera, 47922 Rimini, Italy

**Keywords:** fucoidan, nutritional grade *Macrocystis pyrifera*, low molecular weight fucoidan, inflammatory bowel disease, anti-inflammatory, antioxidant, oxidative stress, DSS-induced colitis

## Abstract

Inflammatory bowel disease (IBD) is a complex and multifactorial disorder characterised by relapsing and remitting inflammation of the intestinal tract. Oxidative stress (OS) is the result of an imbalance between production and accumulation of reactive oxygen species (ROS), which has been associated with inflammatory responses and implicated in the exacerbation of IBD. Fucoidan, a sulfated polysaccharide from brown seaweed, is a well-known anti-inflammatory agent and emerging evidence indicates that fucoidan extracts from *Macrocystis pyrifera* (MPF and DP-MPF) may also modulate oxidative stress. This study investigated the impact of fucoidan extracts, MPF and DP-MPF in a dextran sodium sulphate (DSS)-induced mouse model of acute colitis. 3% DSS was administered in C57BL/6J male mice over a period of 7 days, and MPF and DP-MPF were co-administered orally at a dose of 400 mg/kg body weight. Our results indicated that MPF and DP-MPF significantly prevented body weight loss, improved the disease activity index (DAI), restored colon lengths, reduced the wet colon weight, reduced spleen enlargement, and improved the overall histopathological score. Consistent with the reported anti-inflammatory functions, fucoidan extracts, MPF and DP-MPF significantly reduced the colonic levels of myeloperoxidase (MPO), nitric oxide (NO), malondialdehyde (MDA) and increased the levels of antioxidant enzymes, superoxide dismutase (SOD) and catalase (CAT). In addition, MPF and DP-MPF significantly inhibited levels of pro-inflammatory cytokines in colon-derived tissues. Collectively, our results indicate that MPF and DP-MPF exhibited anti-inflammatory and antioxidant effects representing a promising therapeutic strategy for the cure of IBD.

## 1. Introduction

Inflammatory bowel disease (IBD), including Crohn’s disease (CD) and ulcerative colitis (UC), is a group of chronic, inflammatory gastrointestinal diseases with recurring episodes and unknown etiology [1,2,3]. IBD is typically characterised by colonic or extra-colonic manifestations and is associated with clinical symptoms, including diarrhoea, stomach discomfort, fever, intestinal blockage, and impairment symptoms like blood or/and mucus [4,5,6]. The combination of genetic predispositions and environmental factors leads to the onset of the disease [7,8,9]. Several investigations associated a dysregulated immune response, oxidative stress, and dysbiosis of the gut microbiome with the pathogenesis of IBD [10,11,12,13]. 

The DSS-induced colitis mouse model is one of the most extensively used experimental tools to investigate intestinal inflammation due to the development of symptoms similar to the human IBD, often presenting diarrhoea, rectal bleeding, excessive body weight loss, colon length shortening, loss of goblet cells and ulcerations [14,15]. It is well established that the induction of DSS is toxic to colonic epithelial cells, which ultimately disrupts the epithelial barrier integrity [16,17,18,19]. DSS-induced damage to the mucosal epithelial barrier allows the entry of luminal antigens and microbiota into the mucosa leading to an overwhelming inflammatory response, which is associated with infiltration of immune cells and the production of pro-inflammatory cytokines, including TNF, IL-1β and IL-6 [19,20,21,22]. Furthermore, the overproduction and release of reactive oxygen species (ROS) by leukocytes, macrophages and other immune cells result in persistent oxidative stress, which plays a crucial role in developing DSS-induced intestinal inflammation [19,23,24]. 

The detrimental effects of oxidative stress have been well-established in intestinal inflammation and are believed to be potential etiological and/or triggering factors for IBD [25,26,27]. The infiltration of immune cells into intestinal mucosa results in the upregulation of inflammation-mediated enzymes, including peroxidases, myeloperoxidase (MPO), nitric oxide synthase (NOS), and cyclooxygenases (COXs), which participate in the endogenous generation of free radicals (ROS and RNS) by catalysing the chemical reactions [28,29,30]. The imbalance between pro-oxidants (radicals) and antioxidants contributes to cellular oxidative stress and induces the peroxidation of fatty acids and lipoproteins in cell membranes. Malondialdehyde (MDA), the final product of lipid peroxidation, is highly toxic to cells and tissues [16,31]. In response to the oxidative stress, antioxidant enzymes such as superoxide dismutase (SODs) and catalase (CAT) form the first line of defence against ROS-induced tissue injury by catalysing the free radicals into molecular oxygen and hydrogen peroxide and decreasing the excessive levels of oxygen which is damaging for the cells [32]. This cytoprotective response further promotes the desensitisation against oxidative damage and cytotoxicity [29,33,34,35].

In recent years, numerous drugs, including corticosteroids, nonsteroidal anti-inflammatory medications (NSAIDs), immunosuppressants, and antibodies against cytokines, have been tested to treat IBD. However, these drugs have resulted in variable results with potential side effects, including infections and steroid dependence [36,37,38,39]. Given the need for innovative therapeutic ways to address intestinal inflammation and oxidative damage, novel candidate drugs that synergistically combine the anti-inflammatory and antioxidant actions for treating IBD are desired.

Fucoidans are sulfated polysaccharides isolated from brown seaweeds that exhibit various therapeutic potentials, including antioxidants with anti-inflammatory effects in cell and animal models [40,41,42,43,44,45,46,47,48]. A previous study by Yang Q et al., 2014 reported that fucoidan from *Sargassum horneri* (SF) reduced the levels of malondialdehyde (MDA) and significantly increased the SOD activity in a catfish model, thereby enhancing the antioxidant mechanism and innate immunity [49]. Similarly, Chung et al., 2016, reported that fucoidan induced the levels of SOD through the upregulation of Nrf2 and reduced the oxidative stress in HaCat cells [44]. Furthermore, Chunmei Li et al., 2011, reported that fucoidan reduced the levels of MPO, TNF-α and IL-6 in a rat model of myocardial ischemia-reperfusion (I/R) and regulated the inflammation response via HMGB1 and NF-κB inactivation in I/R-induced myocardial damage [50]. Although various fucoidan species have been elucidated for anti-inflammatory and antioxidant properties in both in vivo and in vitro models, *Macrocystis pyrifera* fucoidan extracts have not been investigated for anti-inflammatory and antioxidant properties, especially in the DSS-induced colitis model. Our previous in vitro studies demonstrated that *Macrocystis pyrifera* extracts possess extensive anti-inflammatory effects in inhibiting the major pro-inflammatory cytokines, TNF-α, IL-1β, and IL-6, in the human THP-1 cell line [48]. 

We therefore hypothesised that *Macrocystis pyrifera* fucoidan extracts (MPF and DP-MPF) might be promising agents in ameliorating the DSS-induced inflammation due to the combination of anti-inflammatory and antioxidant properties. The current study results demonstrated that fucoidan extracts from *Macrocystis Pyrifera* successfully alleviated intestinal inflammation through the inhibition of a vast number of pro-inflammatory cytokines. Additionally, these extracts maintained the redox homeostasis in the colon, which is associated with the consistent reduction of lipid peroxidation (MDA), reduction of MPO activity, reduction in NO generation and the upregulation of antioxidant enzymes, including SOD and CAT. The current study, demonstrated for the first time in this investigation that MPF and DP-MPF exhibit substantial therapeutic potential in reducing the severity of DSS induced colitis in mice by exerting anti-inflammatory and antioxidant properties.

## 2. Materials and Methods

### 2.1. Cell Viability Assay

The cell viability was measured through the MTT assay, as previously described [51]. In short, MTT (3-[4,5-dimethylthiazol-2-yl]-2,5, -diphenyltetrazolium bromide) (Sigma Aldrich, Castle Hill, NSW, Australia) was suspended in phosphate-buffered saline (PBS, pH 7.4) at a final concentration of 5 mg/mL. Raw 264.7 murine macrophage cell lines (1 × 10^5^ cells/well) were incubated at 37 °C, 5% CO_2_ with varying concentrations (50 µg/mL, 100 µg/mL, 200 µg/mL, and 400 µg/mL) of both fucoidan extracts (MPF and DP-MPF) in a U-bottom 96-well tissue culture plate (Thermo Fisher Scientific Inc., Scoresby, VIC, Australia) in a final volume of 100 μL. Following the incubation period of 48 h, MTT reagent (20 μL/well) was added to the cells and incubated for an additional 3 h in the incubator at 37 °C. The cells were then washed three times in PBS. Finally, MTT formazan crystals were dissolved in 100 μL of dimethyl sulfoxide (DMSO), and the absorbance was measured on a microplate reader (Tecan pro-2000 plate reader) at 570 nm. The percentage viability was calculated using the formula: (%) = [100 × (sample abs)/(control abs)].

### 2.2. Cell Culture, Pre-Treatment with MPF and DP-MPF and LPS Stimulation

The raw 264.7 murine macrophage cell lines were obtained from Sigma Aldrich (Castle Hill, NSW, Australia) and were maintained in Dulbecco’s Modified Eagle’s Medium (DMEM) (Sigma) supplemented with 10% (*v*/*v*) heat-inactivated fetal bovine serum FBS (Sigma) and penicillin-streptomycin (100 IU/mL and 1 µg/mL), (Sigma) and incubated at, 37 °C with 5% CO_2_. Cells were seeded at a density of 1 × 10^5^ per well in a round bottom 96 well plate purchased from Thermo Fisher Scientific Inc. (Scoresby, VIC, Australia) and were pre-treated with the desired concentrations of fucoidan extracts (MPF and DP-MPF) (50 µg/mL, 100 µg/mL and, 200 µg/mL) and incubated at 37 °C, 5% CO_2_ for 24 h. Pre-treated cells with fucoidan extracts were stimulated with 1 µg/mL of LPS from Escherichia coli (L4391, Sigma) for 24 h. Cells stimulated with LPS without fucoidan treatment were positive control, while cells without fucoidan and LPS treatments were negative control.

### 2.3. Enzyme-Linked Immunosorbent Assay ELISA

TNF-α, IL-1β, and IL-6 were measured from the collected supernatant from treated cells by ELISA using capture and detection antibodies from Peprotech (Sydney, NSW, Australia) and BD Biosciences (Sydney, NSW, Australia) as per the manufacturer’s instructions. The amount of each cytokine was normalised to the positive control (cells treated with LPS alone). Each experiment was performed at least 3 times. Plate reading and curve fittings were performed on a plate reader (TECAN infinite M200, Männedorf, Switzerland) using iControl software, version 1.10, Männedorf, Switzerland (TECAN Group Ltd.).

### 2.4. DSS-Induced Colitis Model

All the animal work was conducted according to the Australian Code of Practice for Care and Use of Animals for Scientific Purposes (8th Edition 2013). The animal study was approved by the Animal Ethics committee of the University of Tasmania under ethics application number; A18481. C57BL/6 mice (aged 8–10 weeks; average weight 24 g) were maintained under a 12-h day/night light cycle. Mice were given access to standard chow and autoclaved water ad libitum and were initially housed for an acclimatization period of 7 days before being singularly housed and included in any experiments. Mice were randomly divided into four groups (*n* = 12) based on body weight [45]; Healthy control (HC), DSS-induced colitis group (DSS), treatment group with MPF (MPF), and treatment group with DP-MPF (DP-MPF). 3.0% DSS, molecular weight, 30–50 kDa (MP Biomedicals, New South Wales, Australia) was dissolved in autoclaved water to induce acute colitis in all mice except the healthy control group (HC). DSS and fucoidan treatment were started simultaneously, and both continued through the full seven days. Mice’ body weights were recorded daily during the seven days study and were euthanised at the end of the experiment.

### 2.5. Formulation of Fucoidan Extracts (MPF and DP-MPF)

*Macrocystis pyrifera* nutritional grade fucoidan extract (MPF) and low molecular weight depyrogenated *Macrocystis pyrifera* (DP-MPF) fucoidan extract were provided by Marinova Pty Ltd., Cambridge, Hobart, Tasmania, Australia. The batch number for MPF is MPF2017001 and for DPMPF low MW, is SK2001075B. The original algae for both MPF and DPMPF was harvested off the Patagonian coast of Argentina around June 2017. All the fucoidan core products used in our study have a five-year shelf life. The original algae for both MPF and DPMPF were harvested off the Patagonian coast of Argentina around June 2017 & the study was conducted in December 2020. The specific properties of both extracts are described in Table 1 using spectrophotometric assays [52,53] and high-performance size exclusion chromatography, and a gas chromatography based method [53,54]. Fucoidan purity is calculated as the sum of neutral carbohydrates, sulfate, and cations from the hydrolysed isolated polymer. 

Food mash containing fucoidan extracts was prepared as previously described [55]. Briefly, fucoidan extracts (MPF, DP-MPF), powdered chow pellets and 4% sucrose (final concentration *w*/*w*) were mixed in autoclaved water and homogenised to formulate a wet food mash. A food pellet of 3 g containing fucoidan extracts (MPF, DP-MPF) daily dose of 400 mg/kg of mice body weight was served daily in a small dish to the treated animals.

### 2.6. Clinical Parameters and Histological Evaluations

The disease activity index (DAI) was calculated as a composite of the individual scores of stool consistency, signs of gross bleeding on the anus site, and body weight loss, as described previously [56,57]. Furthermore, stool samples from individual mice were tested for occult blood using Hemocult II slides (Beckman Coulter Inc., Brea, CA, USA). All three parameters were recorded daily, and by the end of the experiment, all mice were euthanised via carbon dioxide inhalation for tissue collection. Colons were removed, measured in length, weighed, opened, and cut longitudinally into two halves. One-half of the colon was collected for histopathological evaluations using the swiss roll technique, while the other half was used for protein assays and tissue explant culture. The swiss roll tissues were immersed in 10% *v/v* buffered formalin for fixation for 24–48 h, followed by paraffin embedding. The tissues were sectioned into 4 μm and stained with haematoxylin and eosin (H&E). Leica DM500 microscope (Leica Microsystems, Mannheim, Germany) was used to evaluate the histopathological changes in an investigator-blinded manner as previously described [58,59,60,61].

#### 2.6.1. Measurement of Lipid Peroxidation (MDA), MPO Activity and NO Production

The activity of myeloperoxidase (MPO) was measured using a kit (ab105136) from Abcam, Cambridge, UK. The colon tissues from mice were weighed and homogenised in 4 times the volume of assay buffer from the assay kit. After centrifugation at 13,000× *g* and 4 °C for 10 min, the supernatants were collected, and the activity of MPO was measured according to the manufacturer’s protocol [62]. The values are expressed as U/mg of protein. 

The lipid peroxidation marker, malondialdehyde (MDA) levels, were determined by using a lipid peroxidation calorimetric/fluorometric assay kit (K739, Bio Vision, Melbourne, VIC, Australia), as mentioned previously [63]. In short, mice tissues from the distal colon (DC) were homogenised with the lysis buffer and centrifuged at 13,000× *g* for 10 min. Thiobarbituric acid (TBA) was added to the supernatant and boiled in a water bath at 95 °C for 60 min. The resulting MDA-TBA adduct was quantified calorimetrically at 532 nm. The amount of MDA in the samples was calculated by plotting against an MDA standard (from the kit) calibration curve. The final values were expressed as nmol/mg protein.

The amount of nitrite, a stable by-product of nitric oxide (NO), was measured to calculate the NO generation using the Griess reagent kit (G2930, Promega, Alexandria, VIC, Australia), as described previously [64]. The tissue explants from the colon and the nitrite standards (100, 50, 25, 12.5, 6.25, 3.13, 1.56 and 0 μm) were added to the 96-well plate and incubated as per the manufacturer’s protocol. The sample absorbance was plotted against the nitrite standard curve at 550 nm. The values were expressed as a concentration in μm/gram of tissue.

#### 2.6.2. Superoxide Dismutase (SOD), Catalase (CAT) Assay

The activity of SOD and CAT enzymes was measured from the collected colon tissues using the commercially available kits (ab65354 and ab83464), respectively, from Abcam, Cambridge, UK. In short, the DC tissues from mice were weighed, homogenised, and centrifuged at 1100× *g* for 15 min at 4 °C. The levels of SOD and CAT were measured in duplicates, and the assays were performed according to the manufacturer’s protocol. The SOD activity is presented as the % inhibition of superoxide production by SOD. At the same time, CAT activity is presented in U/mg protein [65,66].

#### 2.6.3. Measurement of Cytokine Levels from Explant Tissue Culture

The excised DC sections were weighed and washed with PBS before being cultured in 12–well plates containing 400 μL/well of Roswell Park Memorial Institute (RPMI) 1640 culture medium (In Vitro Technologies Pty Ltd., Melbourne, VIC, Australia), supplemented with 10% Fetal Bovine Serum (Gibco, Life Technologies Pty Ltd., Melbourne, Australia) and 1% antibiotics solution (containing 10 mg/mL streptomycin and 10,000 U/mL of penicillin; Sigma-Aldrich Pty Ltd., Sydney, NSW, Australia), as previously described in [19,56]. After incubating for 24 h at 37 °C, the supernatants were collected, centrifuged, and analysed for cytokine detection using a Bio-Plex Pro Mouse cytokine 23-plex kit (#M60009RDPD, Bio-Rad Laboratories, Sydney, NSW, Australia) according to the manufacturer’s protocol in a Bio-Plex 200 instrument (Bio-Rad Laboratories, New South Wales, Australia). The data was analysed using the Bioplex Manager software, version 6 (Bio-Rad Laboratories, New South Wales, Australia). The cytokine data are normalised to the weight of tissue explant (gram), and the final values were presented as pg/mL/g.

### 2.7. Statistical Analysis

Statistical analysis was performed using GraphPad Prism version 8.3.0 for Windows (GraphPad Software, San Diego, CA, USA, (www.graphpad.com) accessed on 5 March 2022), and results were expressed as mean ± SD. All the experimental data were analysed by one-way ANOVA, except body weight changes and DAI scores in DSS-induced colitis mice, which were analysed by two-way ANOVA. All ANOVA results were followed by a post hoc analysis using Tukey’s comparison test as appropriate. Results were considered statistically significant when *p* < 0.05.

## 3. Results

### 3.1. Cell Culture

#### 3.1.1. Effect of MPF and DP-MPF on the Viability of Macrophage Cell Line

**Figure 1:**
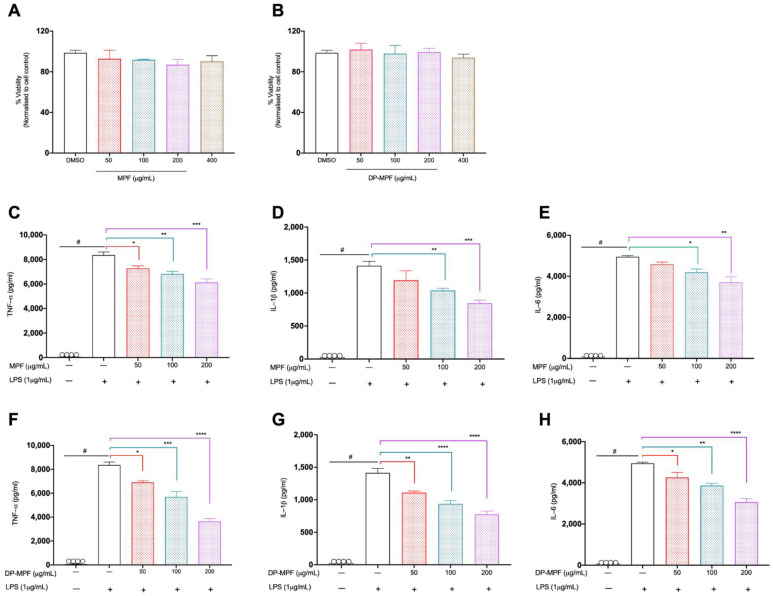
(**A**,**B**). Effects of MPF and DP-MPF on raw 264.7 macrophage cell line viability. Cells were treated with the specified concentrations of fucoidan extract (**A**) MPF and (**B**) DP-MPF for 48 h and assessed by MTT assay. Results expressed as % viability of treated cells vs. vehicle control. (**C**–**H**). Effects of MPF and DP-MPF on pro-inflammatory cytokines, TNF-α, IL-1β, and IL-6 in LPS-stimulated raw 264.7 cell line. Cells were pre-treated with the indicated doses (**C**–**E**) MPF and (**F**–**H**) DP-MPF for 24 h, followed by LPS stimulation for 24 h. TNF-α, IL-1β, and IL-6 were measured from the supernatant using ELISA. Data expressed as means ± SEM. Statistical significance was carried out using one-way ANOVA followed by Tukey’s multiple comparison test (*n* = 3). **^#^** Significant effect HC vs. DSS (*p* < 0.05); * Significant effect treatment vs. DSS (DSS vs. MPF) or (DSS vs. DP-MPF). A *p*-value of <0.05 was considered significant. * *p* < 0.05, ** *p* < 0.001, *** *p* < 0.0001 and **** *p* < 0.00001.

The MTT assay is a typical cell proliferation assay performed to analyse the short-term cytotoxicity of the testing compound in the cells. It is based on the metabolic production of NADPH as a surrogate marker for cytostatic activity and cell death. Murine macrophage cells were treated with different concentrations of MPF and DP-MPF (0, 50, 100, 200, 400 µg/mL) for 48 h (Figure 1A,B). MPF and DP-MPF exhibited no signs of cytotoxicity after 48 h for the treated doses.

#### 3.1.2. Effects of MPF and DP-MPF on LPS Induced TNF-α, IL-1β, and IL-6 in Raw 264.7 Macrophage Cell Line

MPF and DP-MPF effects on pro-inflammatory cytokines (TNF-α, IL-1β, and IL-6) were investigated in the LPS-induced macrophage cell line. MPF and DP-MPF treatment significantly reduced all three cytokine levels in a dose-dependent manner. MPF reduced the levels of TNF-α levels by 13% (*p* < 0.0351), 18% (*p* < 0.0039) and 26% (*p* < 0.0002), IL-1β by 15% (ns), 26% (*p* < 0.0379), 40% (*p* < 0.0026) and IL-6 by 7% (ns), 15% (*p* < 0.0341) and 25% (*p* < 0.0013) at the concentrations of 50 µg/mL, 100 µg/mL, and 200 µg/mL, respectively (Figure 1C–E). Similarly, DP-MPF significantly reduced the levels of TNF-α by 17% (*p* < 0.0197), 31% (*p* < 0.0002) and 56% (*p* < 0.0001), IL-1β by 21% (*p* < 0.0051), 33% (*p* < 0.0002), 45% (*p* < 0.0001) and IL-6 by 13% (*p* < 0.0466), 21% (*p* < 0.0026) and 38% (*p* < 0.0001) at the concentrations of 50 µg/mL, 100 µg/mL, and 200 µg/mL, respectively (Figure 1F–H).

### 3.2. Animal Study

#### 3.2.1. MPF and DP-MPF Improved the Clinical Parameters and Macroscopic Features of DSS-Induced Colitis

**Figure 2:**
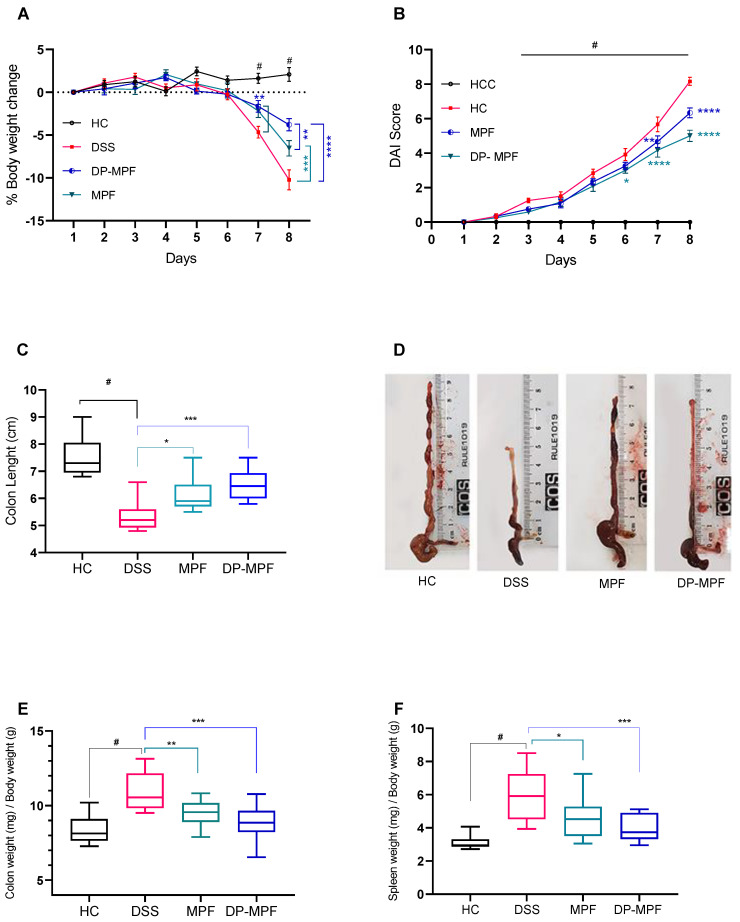
Effect of MPF and DP-MPF on the pathology of dextran sodium sulphate (DSS)-induced acute colitis. (**A**) % body weight change, (**B**) disease activity index (DAI) of healthy controls (HC), DSS, MPF and DP-MPF treated groups. Statistical significance was evaluated using two-way ANOVA followed by Tukey’s post-test. ^#^ Significant effect HC vs. DSS (*p* < 0.05); * Significant effect treatment vs. DSS (DSS vs. MPF) or (DSS vs. DP-MPF). A *p*-value of <0.05 was considered significant. * *p* < 0.05, ** *p* < 0.001, *** *p* < 0.0001 and **** *p* < 0.00001. Data expressed as a mean ± SEM (*n* = 12/group). (**C**) Colon length, (**D**) Colon lengths macroscopic appearance, (**E**) Colon weight and (**F**) Spleen weight, as a mean ± SEM (*n* = 12/group), assessed using one-way ANOVA followed by Tukey’s post-test.

3% of DSS administration for 7 days in mice induced severe clinical symptoms of acute colitis. A significant loss in body weight, diarrhoea and gross bleeding was observed in all the groups except the healthy control (HC) group. Mice from the DSS group consistently recorded body weight loss daily until day 8 (−10.23, ±4%) (Figure 2A). In contrast, DSS-treated mice receiving MPF and DP-MPF demonstrated a significant reduction in weight loss (−6.54, ±3.13% and 3.79, ±2.46%) till day 8 (Figure 2A). Additionally, the disease activity index (DAI), calculated as a collective score of occult blood, stool consistency and body weight loss, was significantly (*p* < 0.0002) increased in DSS treated group from day 3 onwards compared to HC (Figure 2B). In contrast, DSS-treated mice receiving MPF and DP-MPF showed significantly low DAI scores due to reduced occult blood and diarrhoea scores. DP-MPF displayed a significant difference from day 6 (*p* < 0.0123), day 7 for MPF and DP-MPF (*p* < 0.0050, *p* < 0.0001) and day 8 (*p* < 0.0001, *p* < 0.0001), respectively, compared to DSS treated group (Figure 2B). Furthermore, DSS treatment resulted in significant (*p* < 0.0001) shortened colon lengths (5.22 cm, ±0.34) compared to HC (7.82 cm, ±0.79) (Figure 2C,D). In line with the reduced DAI score, MPF and DP-MPF significantly improved the colon lengths (6.12 cm, ±0.63, *p* = 0.0223, and 6.50 cm, ±0.59, *p* = 0.0002) compared to the DSS-treated group (Figure 2C,D). Furthermore, wet colon weight is an important indicator of intestinal inflammation and oedema, calculated as the ratio of colon weight and total body weight (mg/g). DSS treatment significantly increased the wet colon weight in the DSS-treated group (10.96 ± 1.29 mg/g, 24.02%) compared to HC (Figure 2E); however, MPF and DP-MPF treatment significantly reduced the relative wet colon weight with 13.14% and 10.71%, (9.5 ± 0.91 mg/g, *p* < 0.0256 and 9.09 ± 1.46 mg/g, *p* < 0.0003), respectively compared to the DSS group (Figure 2E). Spleen enlargement is another important indicator of intestinal inflammation; DSS treatment resulted in significant enlargement of the spleen (5.90 ± 1.51 mg/g) compared to HC (Figure 2F), while MPF and DP-MPF treatment significantly reduced the relative spleen enlargement (4.57 ± 1.25 mg/g, *p* < 0.0263 and 4.05 ± 0.81, *p* < 0.0009), respectively, compared to DSS treated group (Figure 2F).

#### 3.2.2. MPF and DP-MPF Reduced DSS-Induced Histopathology of the Colon in Acute Colitis

**Figure 3:**
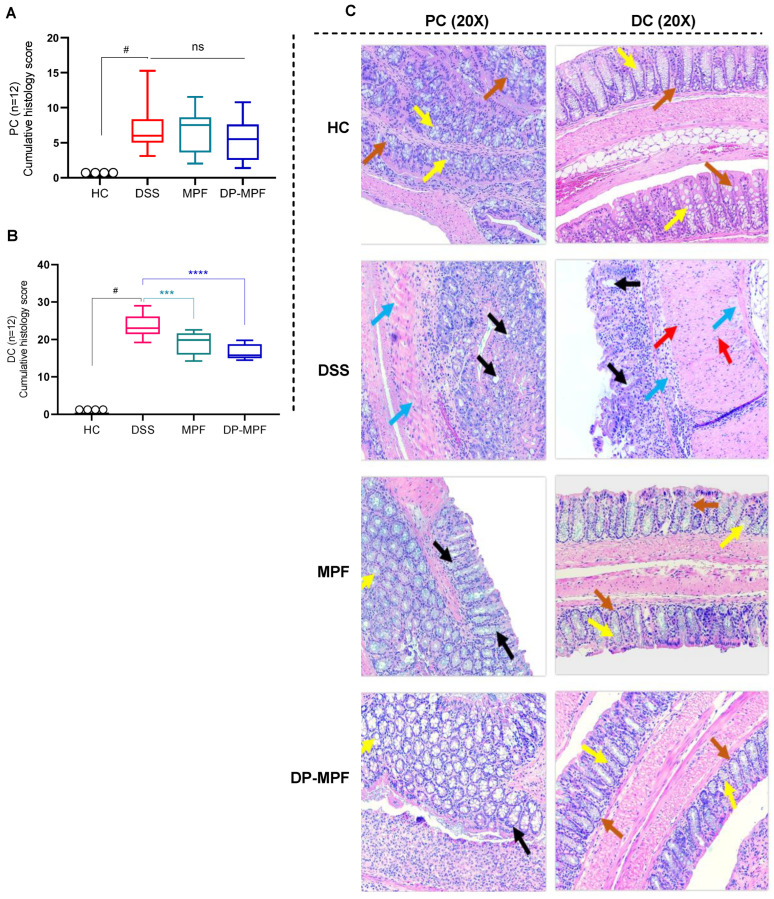
Effect of MPF and DP-MPF on histopathology in DSS-induced colitis. (**A**,**B**) Histopathological scores for each animal were calculated after microscopic analysis of tissue sections from the PC and DC. (**C**) Histological representation of PC and DC sections stained with haematoxylin and eosin (H&E) for healthy controls (HC), DSS-treated mice (DSS), MPF treated mice (MPF), and DP-MPF treated mice (DP-MPF) at 20× magnification. Statistical significance was evaluated using one-way ANOVA followed by Tukey’s post-test. **^#^** Significant effect HC vs. DSS (*p* < 0.05). A *p*-value of <0.05 was considered significant, ns indicates non-significance, *** *p* < 0.001 and **** *p* < 0.0001. Data expressed as a mean ± SEM (*n* = 12/group). Arrows represent crypts/regeneration of crypts (brown), goblet cells (yellow), epithelium surface erosion (black), inflammatory cells infiltration (red) and submucosal oedema (blue).

Haematoxylin and eosin (H&E) stained tissue sections were used to assess the histopathology of the proximal colon (PC) and distal colon (DC). H&E-stained sections of mice from the HC group exhibited no signs of inflammation and damage to the colonic mucosal structure in the stained sections. Alternatively, DSS-treated mice exhibited severe destruction of colonic crypts, epithelial damage, goblet cell loss, submucosal oedema, and inflammatory cellular infiltration in the mucosa, mainly in the DC (Figure 3A–C). This resulted in a high cumulative histology score of (23.18, ±3.48) for DC and (7.82, ±3.39) for PC in the DSS-treated group (Figure 3A,B). In contrast, MPF and DP-MPF treated mice demonstrated evident protection against colonic inflammation with retention of colonic structure, reduced goblet cell loss and reduced infiltration of inflammatory cells (Figure 3C), resulting in a lesser histology score (19.05, ±2.99 and 16.09, ±2.49) for DC and (6.54, ±3.08, and 5.57, ±3.07) for PC, respectively compared to the DSS group (Figure 3A,B).

#### 3.2.3. MPF and DP-MPF Reduced the Oxidative Stress Markers MPO, NO and MDA

**Figure 4:**
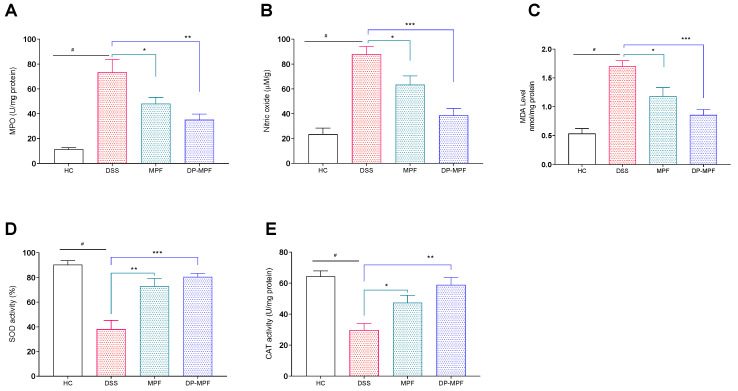
Effect of MPF and DP-MPF on oxidative stress markers and antioxidant enzymes in DSS-induced acute colitis. (**A**) Myeloperoxidase (MPO) activity in DC, (**B**) nitric oxide (NO) concentration (μM)/gram in DC, (**C**) Malondialdehyde (MDA) levels in the DC, (**D**) total SOD activity in the DC representing the reduction of xanthine oxidase activity, expressed as a % inhibition and (**E**) CAT activity in DC. Data expressed as a mean ± SEM (*n* = 5/group). Statistical significance was evaluated using one-way ANOVA followed by Tukey’s post-test, A *p*-value of <0.05 was considered significant, * *p* < 0.05, ** *p* < 0.01 and *** *p* < 0.001. **^#^** Significant effect HC vs. DSS (*p* < 0.05); * Significant effect treatment vs. DSS (DSS vs. MPF) or (DSS vs. DP-MPF).

The protective role of MPF and DP-MPF during oxidative stress conditions was evaluated by assessing the levels of oxidative stress markers such as MPO, NO and MDA in the DC. The DSS treatment significantly increased the levels of these markers (Figure 4A–C), which were significantly reduced by the MPF and DP-MPF treatment (MPO, *p* < 0.0480, 32.55% and *p* < 0.0024, 46.74%), (NO, *p* < 0.0478, 27.91% and *p* < 0.0002, 55.90%) and (MDA, *p* < 0.0197, 30.47% and *p* < 0.0003, 49.55%), respectively, (Figure 4A–C) compared to the DSS group.

#### 3.2.4. MPF and DP-MPF Increased the Levels of Antioxidant Enzymes SOD and CAT

The antioxidant enzymes, including SOD and CAT, are vital in protecting the tissues against oxidative stress. The activity of the SOD enzyme was significantly reduced by the DSS treatment in the DC by 38.10% compared to the healthy mice (HC) (90.33%) (Figure 4D,E). In contrast, MPF and DP-MPF treatment significantly increased the activity of SOD by (*p* < 0.0012, 72.85% and *p* < 0.0002, 80.51%) compared to the DSS group (Figure 4D). In line with SOD activity, DSS treatment significantly reduced the CAT activity (53.68%) in the DC compared to the healthy control (HC). However, MPF and DP-MPF treatment significantly restored the CAT activity (*p* < 0.0498, 37.20% and *p* < 0.0012, 49.41%) (Figure 4E) compared to the DSS group.

#### 3.2.5. MPF and DP-MPF Reduced the Pro-Inflammatory Cytokine Levels in DC Tissue

**Figure 5:**
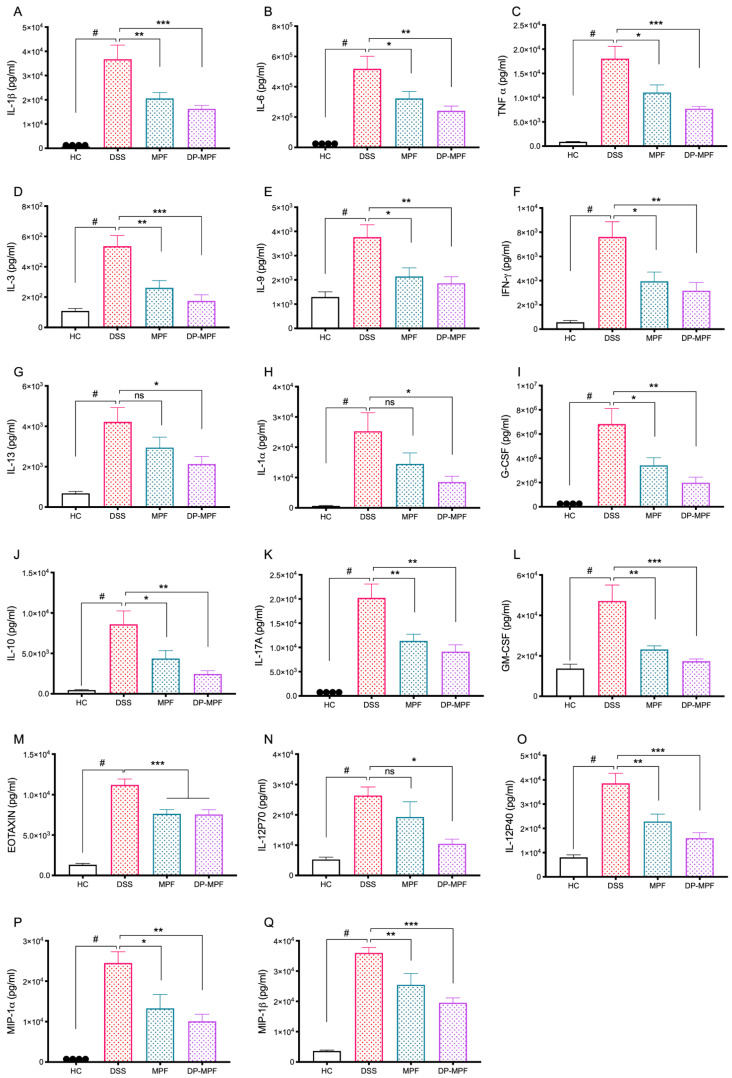
Effect of MPF and DP-MPF treatments on cytokine levels from DC tissues in DSS-induced acute colitis model. (**A**) IL-1β, (**B**) IL-6, (**C**) TNF-α, (**D**) IL-3, (**E**) IL-9, (**F**) interferon-γ (IFN-γ), (**G**) IL-13, (**H**) IL-1α, (**I**) G-CSF, (**J**) IL-10, (**K**) IL-17A, (**L**) GM-CSF (granulocyte colony-stimulating factor), (**M**) Eotaxin, (**N**) IL-12 (P70), (**O**) IL-12 (P40), (**P**) macrophage inflammatory protein (MIP-1α), (**Q**) MIP-1β, Data expressed as a mean ± SEM (*n* = 8/group). Statistical significance was evaluated using one-way ANOVA followed by Tukey’s post-test. A *p*-value of <0.05 was considered significant, non-significant (ns); * *p* < 0.05, ** *p* < 0.01, and *** *p* < 0.00001. **^#^** Significant effect HC vs. DSS (*p* < 0.05); * Significant effect treatment vs. DSS (DSS vs. MPF) or (DSS vs. DP-MPF).

DSS-induced colitis is associated with increased production of pro-inflammatory cytokines and chemokines. To evaluate the effects of MPF and DP-MPF treatment on the production of pro-inflammatory cytokines, the supernatant of tissue explant culture from the DC was investigated for the levels of pro-inflammatory cytokines. The DSS-treated group demonstrated increased levels of pro-inflammatory cytokines. In contrast, MPF and DP-MPF treatment showed significant suppression of pro-inflammatory cytokines and chemokines in DC-derived tissue explants. The levels of TNF-α, IL-1α, IL-1β, IL-3, IL-6, IL-9, IL-10, IL-12 (P40), IL-12 (P70), IL-13, IL-17A, G-CSF (granulocyte-macrophage colony-stimulating factor), GM-CSF (granulocyte colony-stimulating factor), IFN-γ (interferon gamma) were significantly reduced (Figure 5, Table 2). In addition, MPF and DP-MPF also substantially inhibited the production of chemokines, including MIP-1α (macrophage inflammatory protein 1 alpha), MIP-1β (macrophage inflammatory protein 1beta), and Eotaxin in the DC (Figure 5, Table 2). The percentage inhibition for each cytokine by MPF and DP-MPF is calculated and presented in Table 2.

## 4. Discussion

In the present study, we report the important antioxidant and anti-inflammatory properties of fucoidan extracts from *Macrocystis pyrifera* (MPF and DP-MPF) in a mouse model of acute colitis.

Fucoidan has been known for its low cytotoxicity, anti-inflammatory, and antioxidant properties in many experimental models [45,48,67,68].

The cytotoxicity evaluation of the testing compounds is a critical step prior to in vitro and in vivo studies [69,70]. Several studies have reported the minimal cytotoxicity of fucoidan on the viability of several cell lines, including BCBL-1, HL-60, HS-Sultan, K562, NB4, TY-1, THP-1 monocytes, and U937 cell lines [71,72,73,74]. In line with previous findings, we demonstrate that MPF and DP-MPF exhibit no cytotoxicity on murine macrophages up to 400 µg/mL for 48 h.

In the current study, we administered MPF and DP-MPF fucoidan extracts in a DSS-induced mouse model at a dose of 400 mg/kg, which would translate to approximately 2.4 g/day in men (based on a 70 kg body weight) [75]. Oral administered fucoidan up to 6 gm/day in humans is considered safe without any adverse side effects [76], which places the dosage administered in the current investigation into a therapeutic range that might be used in future clinical studies.

The oral administration of DSS in the current study replicated the symptoms of human ulcerative colitis in mice, such as body weight loss, blood in stool, diarrhoea and colon shortening, which is in line with the previous studies [19,45,77]. 

Body weight loss is an essential indicator of the severity of colonic inflammation during ulcerative colitis and is strongly correlated with histopathological alterations [78]. The increased production of pro-inflammatory cytokines such as TNF-α, IL-1β, and IL-6 is reported to have significant involvement in body weight reduction during the UC, as these cytokines are associated with the suppression of appetite neuropeptides [79]. Furthermore, reduced food intake, malabsorption, and excessive loss of fluid resulting from diarrhoea and rectal bleeding further contribute to body weight loss during colitis [80,81,82,83].

Similarly, increased disease activity index (DAI), shortened colon lengths, increased wet colon weight, and increased spleen weight is strongly associated with DSS-induced colitis [77], and our results in the current study have replicated all these macroscopic symptoms in the DSS treated group.MPF and DP-MPF treatment significantly improved the body weight, reduced the overall DAI score, reduced the wet colon weights, reduced the spleen weight, and improved the colon lengths, suggesting the protective effect of both MPF and DP-MPF in DSS-induced colitis.

DSS-induced colitis is strongly associated with epithelial changes, such as crypt shortening and branching, reduced crypt density, excessive infiltration of inflammatory cells to the mucosa and goblet cell loss. Our histological investigations in DSS-treated mice mirrored these structural alterations and colonic damage [19,84,85]. MPF and DP-MPF treatment significantly reduced the inflammatory cell infiltration in the intestinal mucosa and restored the colonic structures, including the crypt structure, crypt lengths and partially reestablished the goblet cell loss. Furthermore, MPF and DP-MPF-treated mice ameliorated the DSS-induced intestinal inflammation and damage, evidenced by the low cumulative score in the DC.

Reactive oxygen species (ROS) and reactive nitrogen species (RNS) are the intermediate products of cellular metabolism and play vital roles in maintaining signal transduction and intracellular homeostasis [27,29]. However, during intestinal inflammation, an imbalance between the production and elimination of ROS leads to oxidative stress and tissue damage [27,28,86]. The mucosal infiltration of immune cells such as neutrophils and monocytes contribute to cellular oxidative stress by distinctively secreting the myeloperoxidase (MPO) enzyme that can produce powerful oxidants [87,88]. In addition, other radicals, including nitric oxide (NO), further intensify cellular oxidative stress, leading to lipid peroxidation. Lipid peroxides adversely alter the membrane structure and function, generating highly reactive toxic products such as Malondialdehyde (MDA), one of the final products of lipid peroxidation, that reacts with proteins and DNA, compromising their regular activity and induces tissue damage [31,89,90,91]. Previous studies have reported that DSS-induced colitis is associated with increased levels of MDA, MPO and NO and our results from current research have replicated these findings [88,92,93]. The DSS-treated group has demonstrated a significant increase in MPO, MDA and NO levels. On the contrary, MPF and DP-MPF treatment significantly reduced all three oxidative stress markers, suggesting an anti-oxidative effect of these extracts against oxidative stress markers.

Furthermore, the cellular machinery counteracts oxidative stress through Nrf2-mediated upregulation of the antioxidant system [94,95]. During oxidative stress, the Nrf2 transcription factor translocates to the nucleus and binds to the promotor regions of the genes that encode antioxidant enzymes such as SOD and CAT [96,97]. SOD and CAT are primary enzymes against lipid peroxidation and are the first line of defense against cellular oxidative stress and tissue damage. SOD and CAT neutralise the free radicals into molecular oxygen and hydrogen peroxide, reducing the extreme oxygen levels, which is detrimental to the cells and tissues [32,98]. In the current study, DSS treatment significantly reduced the SOD and CAT levels due to excessive oxidative stress in DSS treated group. However, 7 Days of MPF and DP-MPF treatment significantly restored the antioxidant enzyme levels of SOD and CAT in the treatment groups. Recent literature [67] reported the antioxidant activity of fucoidan in a mouse model of liver injury and human hepatocyte HL-7702 cell line that involved the upregulation of Nrf2 derived SOD and CAT activity and downregulation of MDA levels from inhibiting the oxidative stress Acetaminophen-induced liver injury [67]. Our results suggest that MPF and DP-MPF exhibit strong antioxidant activity by reducing the cellular oxidative stress markers (MDA, MPO, NO) and promoting the antioxidant enzymes (SOD, CAT) mediated by the Nrf2 transcription factor.

The progression of IBD is accompanied by elevated levels of pro-inflammatory cytokines and a decrease in anti-inflammatory cytokines [99,100]. An aberrant secretion of pro-inflammatory cytokines cascade in response to mucosal injury in the lamina propria results in intestinal inflammation [5,101,102]. The DSS-induced colitis mouse model is primarily a macrophage/Th1/Th17 driven inflammatory model with increased levels of pro-inflammatory cytokines such as TNF-α, IL-1β and IL-6. TNF-α is a hallmark of inflammation, secreted by activated macrophages, monocytes and differentiated T helper 1 cells [103,104]. TNF-α exerts its pro-inflammatory effects through increased production of IL-1β and IL-6, expression of adhesion molecules and inhibition of apoptosis [22,105]. TNF-α interaction with type 2 receptor leads to an inflammatory cascade through NF-κB activation. NF-κB is a well-known transcription factor that encodes for numerous pro-inflammatory genes downstream and plays a crucial role in inflammatory signalling pathways [106,107,108]. Likewise, IL-1β is also associated with the initiation of the inflammatory cascade and is known to activate caspase-1, which causes programmed cell death [109,110]. At the same time, IL-6 promotes the differentiation of diverse immune cell types, including T cells via STAT3 signalling pathway [111,112]. IL6/STAT3 induction of anti-apoptotic factors such as Bcl-2 and Bcl-xL lead to mucosal T cell accumulation and apoptotic resistance, ultimately leading to chronic inflammation [22,111,112,113]. Furthermore, IL 6, along with TNF-α, supports the Th17 cell development and induces the production of IL-17. IL-17 then synergise with TNF-α and IL-6 to enhance the production of pro-inflammatory mediators during intestinal inflammation [22,114,115,116]. TNF-α, IL-1β, and IL-6 are, therefore, key targets due to their predominant role in intestinal inflammation [22,114,117,118]. This study initially tested MPF and DP-MPF extracts for their anti-inflammatory activity in LPS-induced macrophage cell lines. LPS stimulation elevated the levels of TNF-α, IL-1β and IL-6 in murine macrophages. However, pre-treatment with MPF and DP-MPF significantly suppressed the LPS-induced TNF-α, IL-1β, and IL-6 levels in a dose-dependent manner. Similarly, in the mice model, DSS treatment exhibited elevated levels of TNF-α, IL-1β and IL-6 in mice colon tissues. While orally administered MPF and DP-MPF mice demonstrated significant suppression of TNF-α, IL-1β and IL-6 levels in the treatment groups. These anti-inflammatory effects of fucoidan are in line with the recent in vitro studies, where MPF and DP-MPF significantly reduced TNF-α, IL-1β and IL-6 levels in LPS induced human THP-1 cells [48]. Additionally, lean et al., 2015 [45] reported that Fucus polyphenol complex and depyrogenated fucoidan (DPF) extracts from *Fucus vesiculosus* downregulated several cytokines, including TNF-α, IL-1β and IL-6 in a DSS-induced colitis model [45]. The results of the current study are consistent with the previous findings [45,47,48,119]. In addition, MPF and DP-MPF also suppressed increased levels of IL-1α, IL-3, IL-9, IL-12 (P40), IL-12 (P70), GM-CSF, and G-CSF, which are associated with the differentiation and recruitment of monocytes to macrophages into the lamina propria during intestinal inflammation [120,121]. IL-9, which is also upregulated in response to DSS, is tightly correlated with IL-13 and is associated with the hyperplasia of intestinal Paneth cells via upregulation of IL-13 expression [122,123]. Additionally, IL-12 subunits are associated with the activation of Janus kinase 2 (JAK2) and tyrosine kinase 2 (TYK2), which consequently activate the signal transducer and activator of transcription STAT4. The activation of STAT4 is essential for IFN-γ induction and Th1 differentiation [124]. IFN-γ upregulation is associated with the vascular barrier dysfunction resulting in the infiltration of commensal antigens in the intestinal mucosa which plays an important role in the pathophysiology of IBD [121,125]. The elevated levels of IL-1α, IL-1β, IL-3, IL-6, IL-9, IL-12 (P40), IL-12 (P70), IL-17, GM-CSF, and G-CSF in response to DSS treatment in the current study, supports the strong involvement of macrophage activation in the DSS induced colitis model. While results from the treatment groups suggest that MPF and DP-MPF exert strong anti-inflammatory effects possibly via blocking the macrophage maturation and activation, subsequently reducing the cytokine levels both in vitro and in vivo DSS colitis model. Similarly, IFN-γ levels were reduced by MPF and DP-MPF treatment, correlates with the reduced levels of IL-12 by MPF and DP-MPF treatment in this study.

IL-10 is an anti-inflammatory cytokine reported to have elevated levels in colitis patients, and our current study has replicated these results in DSS-treated mice [126,127,128,129]. These elevated levels could be the compensatory mechanism to counterbalance the significant intestinal inflammation. Especially in the context of MPF and DP-MPF inhibition of pro-inflammatory response, a decline in IL-10 levels in the current study does not seem surprising. 

In addition to cytokines, our results align with the previous in vivo and clinical findings that reported the upregulated levels of chemokines such as Eotaxin, MIP-1α and MIP-1β during IBD [130,131]. Eotaxin is a potent chemoattractant for eosinophils and has been reported to be overexpressed during intestinal inflammation. Increased infiltration of eosinophils in intestinal mucosa strongly correlates with diarrhoea, tissue destruction, fibrosis formation and strictures [132,133]. While MIP-1α and MIP-1β are potent chemoattractants for monocytes/macrophages, they also induce neutrophil infiltration into intestinal mucosa, resulting in oxidative stress and tissue damage [134]. MPF and DP-MPF treatment significantly reduced the levels of these upregulated chemokines in response to DSS treatment. Chemokines are small proteins (7–10 kDa) that govern the massive recruitment of inflammatory cells to the sites of inflammation due to aberrant chemoattraction for leukocytes [135] and bind to the GPCRs to initiate several signal transduction pathways and induce cellular response to attract the recruitment of inflammatory cells [136]. Fucoidan has been reported to exhibit inhibitory effects on the GPCRs associated with Ca^2+^ in HeLa cells, human umbilical vein endothelial cells and astrocytes cell models. [137]. Here, we demonstrate that MPF and DP-MPF suppression of chemokines, including Eotaxin, MIP-1α and MIP-1β in our study could be due to the inhibition of GPCRs receptors by MPF and DP-MPF, and these extracts could possibly function as antagonists for GPCRs resulting in inactivation of cell signalling pathways and signal transductions that eventually lead to reduced immune response. Especially, DP-MPF is a low molecular weight fucoidan extract with molecular weight ranging from 5–30 kDa, compared to chemokine protein size (7–10 kDa). Further studies investigating the interaction between fucoidan and GPCRs would help in understanding the underlying molecular mechanisms. Nevertheless, it should be mentioned that low molecular extracts have shown to be efficient anti-inflammatory agents in previous studies due to their small molecular size, excess to the receptor sites and better absorption through the cell surface [48].

## 5. Conclusions

The excessive oxidative stress during colitis induces colonic damage and stimulates the inflammatory immune cells that release the pro-inflammatory cytokines and chemokines, resulting in persistent inflammation [19,25]. In our investigations, fucoidan extracts MPF and DP-MPF initiated and regulated cellular events that significantly reduced oxidative stress and inflammation in DSS-induced colitis. Notably, the disease severity index, macroscopic and histological findings strongly correlate with the reduction of pro-inflammatory cytokines, reduction in the oxidative stress markers and elevated levels of SOD and CAT antioxidant enzymes. *Macrocystis pyrifera* extracts MPF and DP-MPF have been shown for the first time to possess strong anti-inflammatory and antioxidant properties in the DSS-induced colitis model. Further preclinical studies are needed to elucidate the underlying molecular mechanisms regulated by MFP and DP-MPF in animal models of DSS-induced colitis.

## Figures and Tables

**Table 1:** The composition and carbohydrate profile of fucoidan extracts (MPF and DP-MPF).

Code	Species		Origin		Description	Purity	
MPF	*Macrocystis pyrifera*		South American		Nutritional grade extract	≥85% purity	
DP-MPF	*Macrocystis pyrifera*		South American		Depyrogenated extract, (5–30 kDa)	≥85% purity	
Code	Total Carbohydrates (%)		Uronic Acid (%)	Polyphenols (%)	SO_4_ (%)	Cations (%)	Peak MW (kDa)
MPF	51.1		6.1	<2.5	25.7	7.9	66.0
DP-MPF	54.6		7.1	<2.5	19.7	10.8	17.4
Code	Fucose	Xylose	Mannose	Galactose	Glucose	Arabinose	Rhamnose
MPF	66.2	3.1	5.9	15.6	3.9	2.3	3.0
DP-MPF	62.9	4.5	8.1	14.3	6.9	0.4	3.0

**Table 2:** Percentage inhibition of DC-derived pro-inflammatory cytokines and chemokines by MPF and DP-MPF.

Cytokine	MPF	DP-MPF
	% Change (*p*-Value)	
TNF-α	−38.72 (0.0154)	−57.27 (0.0004)
IL-1α	ns	−69.02 (0.0195)
IL-1β	−43.98 (0.0084)	−55.60 (0.0009)
IL-3	−59.44 (0.0042)	−76.00 (0.0002)
IL-6	−37.91 (0.0474)	−53.45 (0.0038)
IL-9	−43.09 (0.0194)	−50.50 (0.0055)
IL-10	−49.14 (0.028)	−71.45 (0.0012)
IL-12 (P40)	−40.81 (0.0051)	−58.55 (0.0001)
IL-12 (P70)	ns	−60.43 (0.0108)
IL-13	ns	−49.44 (0.0297)
IL-17A	−43.80 (0.0087)	−54.75 (0.0011)
Eotaxin	−31.93 (0.001)	−32.68 (0.0008
G-CSF	−44.98 (0.0223)	−68.10 (0.0026)
GM-CSF	−50.83 (0.002)	−63.19 (0.0002)
IFN-γ	−48.08 (0.0232)	−58.31 (0.0051)
MIP-1 α	−45.74 (0.0230)	−58.84 (0.0030)

The supernatant from the tissue explant culture of the DC was measured for cytokine levels. The percent inhibition (%) is calculated for each cytokine versus the DSS colitis group. Not significant (ns); interleukin (IL); macrophage inflammatory protein (MIP); granulocyte colony-stimulating factor (G-CSF); granulocyte-macrophage colony-stimulating factor (GM-CSF); interferon-γ (IFN-γ); tumor necrosis factor-α (TNF-α), *Macrocystis pyrifera* (MPF) and depyrogenated *Macrocystis pyrifera* (DP-MPF).

## Data Availability

The data presented in this study are available on request from the corresponding author.
